# Microplastics in neonates: an overlooked cost of parenteral nutrition

**DOI:** 10.3389/fpubh.2026.1767555

**Published:** 2026-04-07

**Authors:** Debo Xu, Zhenyu Liang, Qiaohuan Yang, Lin Li, Huiyi Li, Chuming You, Qiong Meng, Wei Zhou

**Affiliations:** Department of Pediatrics, The Affiliated Guangdong Second Provincial General Hospital of Jinan University, Guangzhou, China

**Keywords:** dose–response relationship, iatrogenic exposure, microplastics, neonates, parenteral nutrition

## Abstract

**Introduction:**

Microplastics (MPs) are emerging contaminants that raise concerns due to their potential health effects on vulnerable neonates who may be exposed to medical devices.

**Methods:**

This prospective study investigated the presence of MPs in neonatal blood in relation to parenteral nutrition (PN) duration. A total of 12 neonates were categorized into long-term (>14 days), short-term (3–7 days), and non-exposed control groups. MPs were analyzed using confocal micro-Raman spectroscopy.

**Results:**

Polyethylene accounted for 95.65% of particles detected in all blood samples. MP abundance was significantly higher in the long-term PN group compared to both short-term and control groups (*p* < 0.05), demonstrating a dose–response relationship with PN duration. Notably, larger MPs (>60 μm) were found exclusively in the long-term group.

**Discussion:**

These preliminary findings, derived from a small sample cohort, suggest that the PN system may serve as a potential iatrogenic source of MPs for neonates.

**Clinical trial registration:**

NCT07326761.

## 1 Background

Microplastics (MPs), defined as plastic particles smaller than 5 mm in diameter—a criterion established by the U.S. National Oceanic and Atmospheric Administration (NOAA) and now the most widely accepted standard—exist in various forms, including fragments, fibers, films, pellets, and foams. Due to their minute size, they are often referred to as “PM2.5 in the ocean” (1–3). MPs are virtually ubiquitous, contaminating water bodies, soil, air, and the food chain, with detections reported in sea salt, seafood, drinking water, and even honey (4–5).

In recent years, advancements in analytical techniques have led to the detection of MPs in the human blood, lungs, placenta, and breast milk, confirming their potential for systemic distribution and accumulation in the human body (6–7). This discovery has raised profound concerns among the public and the scientific community regarding their long-term health impacts, particularly for the most vulnerable population—neonates.

Neonates, particularly those hospitalized due to prematurity, low birth weight, or severe intestinal dysfunction, are exposed to conditions that differ significantly from the general population. In the neonatal intensive care unit (NICU), life support and nutritional supply predominantly rely on plastic medical devices, such as intravenous infusion bags, tubing, syringe pumps, and ventilator circuits (8–9). These devices may contain intentionally added MPs or release secondary MPs through physical abrasion and chemical degradation during use (10–11).

Crucially, for critically ill neonates unable to tolerate enteral feeding, total parenteral nutrition (PN) serves as their sole vital source of nutrition. However, the entire “plastic pathway” constituting the PN delivery system represents a potential and continuous source of MP exposure (10). Studies have shown that infusion solutions can become contaminated with detectable MP particles after passing through plastic equipment (12). This implies that neonates receiving intravenous nutrition may be subjected to a unique exposure model of “iatrogenic, continuous microplastic infusion.”

Despite this concern, there remains a lack of research on the internal MP burden in this high-risk group, particularly regarding exposure assessments based on PN duration. The majority of existing studies rely solely on single-point biomonitoring, which does not adequately capture the dynamics and cumulative effects of exposure.

Therefore, this study aims to systematically evaluate, for the first time, the dose–response relationship between PN duration and MP load in neonatal blood by constructing a gradient-exposure cohort. This will clarify the potential MP exposure risk associated with PN as a clinically essential therapy, thereby providing critical scientific evidence to identify high-risk infants and to improve medical device material safety.

The majority of neonatal biomonitoring studies have been based on *in vitro* experiments or random urine samples collected at admission or discharge (13), failing to account for the temporal variability of exposure. Moreover, there is a scarcity of research on the internal MP burden in neonates, particularly regarding quantitative data in their blood.

Consequently, this study aims to assess the current state of MP exposure among neonates during their NICU hospitalization. Through a meticulously designed gradient-exposure cohort, this study seeks to systematically evaluate and compare the MP load in the blood of neonates receiving long-term PN for the first time.

## 2 Materials and methods

### 2.1 Study population and grouping

This prospective observational study was conducted in the NICU of Guangdong Second Provincial General Hospital, Jinan University, in 2025. A total of 12 neonates were enrolled. All mothers of the subjects had no underlying diseases such as heart disease, chronic kidney disease, mental illness, hypertension, diabetes mellitus, gestational hypertension, or gestational diabetes. Their demographic information was recorded by researchers through face-to-face interviews. Written informed consent was obtained from all participants’ guardians, who also completed questionnaires.

A total of 12 neonatal blood samples, 12 breast milk samples, and 12 formula milk samples (from bottles) were collected. To precisely evaluate the dose–response relationship between intravenous nutrition duration and MP load, neonates providing blood samples were divided into the following three groups:

1. Long-term exposure group (*n* = 4): This group comprised of preterm infants who required total parenteral nutrition (TPN) support for >14 days due to severe feeding intolerance, necrotizing enterocolitis, short bowel syndrome, or other severe intestinal diseases.
2. Short-term exposure group (*n* = 4): This group comprised of preterm infants who received TPN support for 3–7 days due to early postnatal adaptation issues (e.g., respiratory distress and transient feeding intolerance) and had successfully transitioned to full enteral nutrition at least 48 h before blood sample collection. This group represents common form of transient iatrogenic exposure in the NICU.
3. Control group (*n* = 4): This group comprised of healthy term neonates with a gestational age ≥37 weeks at birth. This group received no intravenous nutrition support or planned infusion therapy and was used to establish baseline MP levels without relevant iatrogenic exposure.

The study strictly adhered to the ethical principles of the Declaration of Helsinki, and the protocol was approved by the Medical Ethics Committee of Guangdong Second Provincial General Hospital, Jinan University.

### 2.2 Interview content

Through interviews, researchers collected demographic information on the neonates and their mothers, including maternal education level (high school or below, bachelor’s degree, postgraduate or above), annual per capita income (<100,000 Chinese yuan [CNY]; 100,000–200,000 CNY; >200,000 CNY), occupation (government official, professional/technical, general office clerk, commercial service, or other), parity (primipara or multipara), conception method (spontaneous or assisted reproductive technology), birth outcome (preterm or term), delivery mode (vaginal delivery or cesarean section), neonatal sex (male or female), and neonatal weight. The details are presented in Table 1.

**Table 1:** Demographic characteristics of pregnant women and neonates in this study (*n* = 12).

Characteristics	Categories	*N* (%)
Education	High school and below	2 (16.6)
	Bachelor’s degree	6 (50)
	Postgraduate and above	4 (33.4)
Occupation	Government official	0 (0)
	Professional/technical	2 (16.6)
	General office	7 (58.3)
	Business services	2 (16.6)
	Others	1 (8.3)
Annual income per capita (CNY)	<100 thousand	3 (25)
	100–200 thousand	6 (50)
	>200 thousand	3 (25)
Parity	Primipara	5 (41.7)
	Multipara	7 (58.3)
Mode of conception	Natural conception	7 (58.3)
	Assisted reproduction	5 (41.7)
Birth outcome	Preterm	6 (50)
	Full-term	6 (50)
Mode of delivery	Vaginal delivery	7 (58.3)
	Cesarean delivery	5 (41.7)
Sex of newborns	Male	4 (33.3)
	Female	8 (66.7)
Newborn weight (g), median (IQR)	–	2,565 (2,090; 3,020)

### 2.3 Sample collection

Samples were collected by trained pediatricians and pediatric nurses. After delivery, neonatal blood was drawn using a non-plastic syringe and immediately transferred into a glass anticoagulant tube (KERONG, 3 mL; Chengdu, China). Breast milk and formula milk from bottles were collected by trained nurses or family members and promptly placed in sample containers.

To avoid contamination, all consumables that came into contact with the samples during collection were non-plastic. All samples were stored at −80 °C under strict anonymization.

### 2.4 Microplastic analysis

#### 2.4.1 Preparation before experiment

A total of 5 mL of the sample was transferred to a glass bottle, 10 mL of concentrated nitric acid was added for acidolysis, and the mixture was placed in a water bath at 50 °C for 3 days.

Finally, 15 mL of acidolysis solution was filtered onto a silicon film with a pore size of 1 μm, and the silicon film was sent to a confocal micro-Raman spectrometer for detection.

The test tubes, beakers, and pipettes used during the procedure were all glass products and did not come into contact with plastic products.

A blank control sample was prepared by rinsing a blank tube with 5 mL of purified water, transferring the solution to a glass bottle, and performing the same pretreatment procedure as for the samples.

The instrument was tested with a standard prior to analysis to ensure normal operation.

#### 2.4.2 Experimental method

The samples were transferred to glass vials. After a period of digestion, the digestate was obtained. It was then filtered through a membrane for detection using confocal micro-Raman spectroscopy.

All laboratory ware used during the process (test tubes, beakers, pipettes, etc.) was made of glass and did not come into contact with plastic products.

When monochromatic light irradiates a transparent sample, most of the light is transmitted, while a small portion is scattered in various directions by the sample molecules. When photons and sample molecules undergo an inelastic collision, the energy of the scattered light differs from that of the incident light, resulting in changes in both the frequency and direction of the light. This type of light scattering is called Raman scattering.

Raman scattering occurs due to the energy exchange between photons and molecules, altering the photon’s energy. Molecular structures can be resolved by analyzing Raman shift spectra, a method particularly suitable for analyzing non-polar bonds (e.g., C–C backbone) and crystalline materials.

Combined with confocal microscopy, point-by-point scanning generates high-resolution chemical images, enabling precise localization of MP composition and distribution (see Tables 2–4 for materials and instrument parameters used in microplastics analysis in this study and Raman spectrum library).

**Table 2:** Materials used in microplastics analysis in this study.

Name	Manufacturer	Country
Nitric acid	Merck Life Sciences	Germany
Mili-Q ultrapure water machine	MILLIPORE	USA
PE (polyethylene) standard	Jiangsu Zhichuan Technology Co., Ltd.	China
Beaker	Sichuan Shu Bo Co., Ltd.	China
Test tube	Sichuan Shu Bo Co., Ltd.	China
Glass pipette	Tianbo Glass Instrument Co., Ltd.	China
Oscillating water bath	Shanghai Jinghong Experimental Equipment Co., Ltd.	China
Silicon film	Smart membranes	Germany
Micro-Raman Spectrometer-DXR3xi	Thermo Fisher Scientific Corporation	USA

**Table 3:** Instruments and parameters used in microplastics analysis in this study.

Instrument	Parameter
Instrument	Thermo Micro-Raman Imaging System DXR3xi
Wavelength scanning range	600–3,200
Instrument lens	50 × telephoto
Laser power	6 mW
Exposure time	0.05 s
Scan count	5
Data processing software	OMNICxi

**Table 4:** Raman spectrum library used for microplastics analysis in this study.

Order number	Database name
1	Aldrich Linked Raman
2	Raman Imaging Microscope Sample Library
3	Raman Sample Library
4	Organics by Raman Sample Library
5	Microplastics Library

### 2.5 Statistical analysis

All data analyses were performed using SPSS Statistics 26 (IBM, USA), with statistical significance set at a two-sided *p*-value of <0.05.

Normality (Shapiro–Wilk test) and homogeneity of variance were first assessed for continuous data. Normally distributed variables are described as mean ± standard deviation, non-normally distributed variables (primarily MP concentration data) are expressed as median (interquartile range [IQR]), and categorical data are expressed as frequency (percentage).

For comparisons of baseline characteristics among the three groups, one-way ANOVA was used for normally distributed continuous variables, the Kruskal–Wallis H test for non-normally distributed continuous variables, and Fisher’s exact test for categorical variables.

Some graphs were generated using GraphPad Prism 9.0 (GraphPad Software, USA), with data verified for accuracy. Parts of the statistical analysis were also performed using R (version 4.4.1). Some figures were created using the ggplot2 package.

Correlation coefficients were calculated using the cor.test() function in R, and the results of hypothesis testing were obtained using the wilcox.test() function.

## 3 Results

### 3.1 Participant characteristics

The baseline characteristics of the study participants are summarized in Table 1. Among the mothers, spontaneous conception and assisted reproductive technology accounted for seven (58.3%) and five (41.7%) cases, respectively; five (41.7%) were primipara.

The number of term and preterm births was equal, each comprising six cases (50%). The median birth weight of the neonates was 2,565 g.

### 3.2 Microplastics in neonatal blood, breast milk, and bottle-feeding samples

As shown in Table 5, a total of 107 MP particles were detected. Among these, 92 particles were found in neonatal blood samples, and 15 particles were detected in bottle-feeding samples. MPs were detected in all neonatal blood samples, while 6 of 12 bottle-feeding samples tested positive for MPs; no MPs were detected in any breast milk samples.

**Table 5:** Microplastic particle content in the 12 neonatal blood, breast milk, and bottle-feeding samples.

Participants	Frequency of microplastic particles (particles)			Samples’ dry weight (g)			Abundance (particle/g)		
	Neonatal blood	Breast milk	Bottle milk	Neonatal blood	Breast milk	Bottle milk	Neonatal blood	Breast milk	Bottle milk
Num.1	1	–	1	0.8589	0.6482	0.5493	1.16	–	1.82
Num.2	1	–	–	0.7947	0.4387	0.6873	1.25	–	0
Num.3	1	–	3	0.7731	0.5639	0.6937	1.29	–	4.32
Num.4	3	–	1	0.8352	0.4729	0.8739	3.59	–	1.14
Num.5	5	–	2	0.9216	0.8745	0.6483	5.42	–	3.08
Num.6	6	–	2	0.8694	0.8430	0.7629	6.90	–	2.62
Num.7	1	–	–	0.8029	0.7832	0.5729	1.24	–	0
Num.8	4	–	–	0.7976	0.6733	0.6382	5.01	–	0
Num.9	9	–	2	0.8406	0.8473	0.4291	10.70	–	4.66
Num.10	12	–	3	0.7931	0.9478	0.4739	15.13	–	6.33
Num.11	20	–	1	0.8427	0.6782	0.5930	23.73	–	1.68
Num.12	29	–	–	0.7493	0.5739	0.7893	38.70	–	0
Median (IQR)	4.5 (1, 10.5)	–	1 (0, 2)	–	–	–	5.22 (1.27, 12.91)	–	1.75 (0, 3.70)
Total	92	–	15	–	–	–	–	–	–
*p*-value^a^							0.006^b^		

^a^: the Kruskal–Wallis H test ^b^: *p*< 0.05.

The median MP abundance in neonatal blood was 5.22 particles/g (range: 1.16–38.70 particles/g), compared to 1.75 particles/g (range: 0–6.33 particles/g) in bottle-feeding samples. The difference in MP abundance between these two sample types was statistically significant (*p* = 0.006).

### 3.3 Characteristics and particle diameter of microplastics in neonatal blood

**Figure 1:**
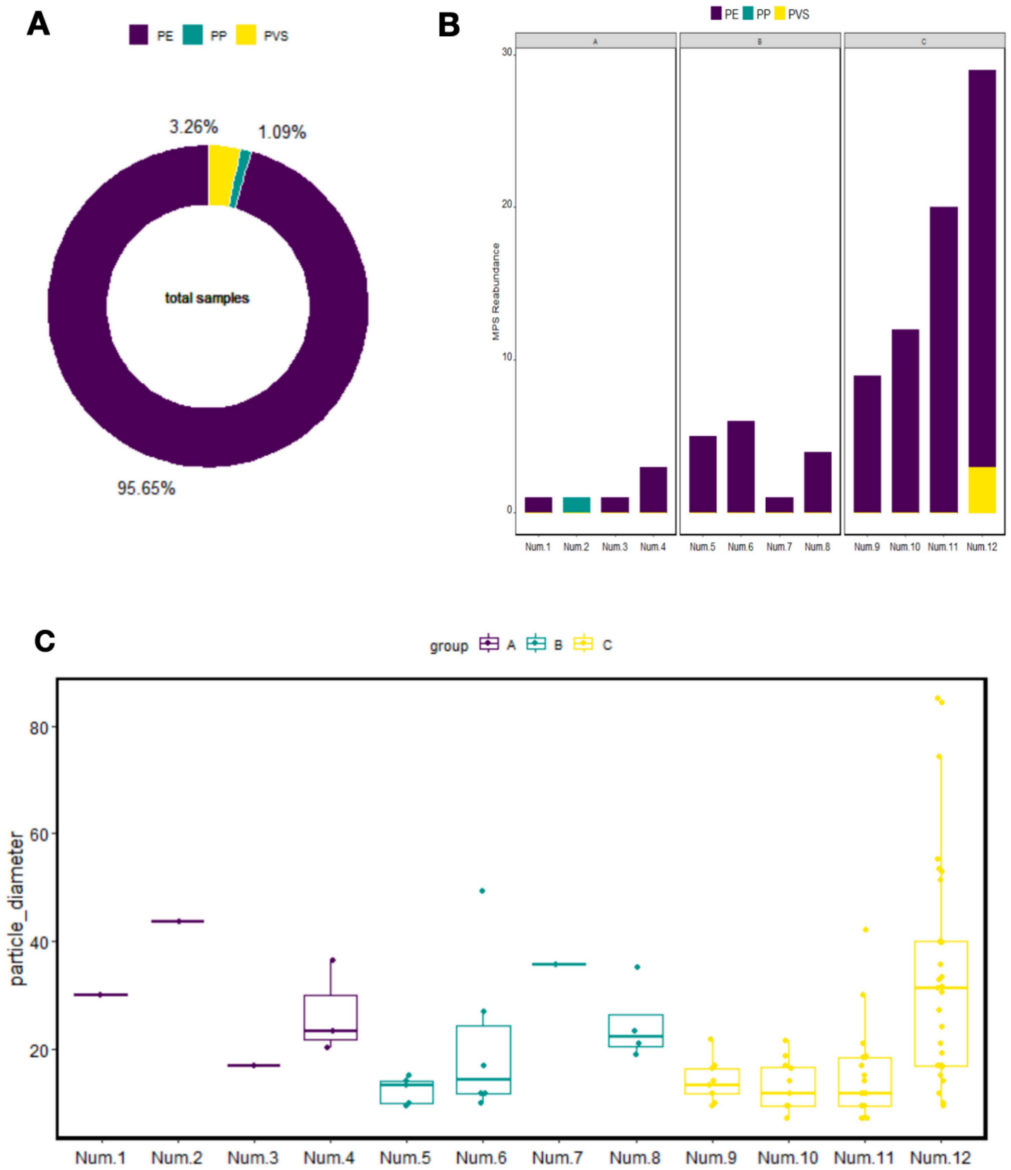
Ring chart of total microplastic ratios across all samples **(A)**, composition of microplastics in individual samples **(B)**, and particle size distribution of microplastics in individual samples **(C)**. PP, polypropylene; PE, polyethylene; PVS, polyvinyl chloride.

The type and frequency distribution of the total MPs identified in neonatal blood samples are presented in Figure 1A. The characteristics of MPs identified in each individual neonatal blood sample are shown in Figure 1B. The particle size distribution of MPs identified per sample is detailed in Figure 1C. Corresponding numerical data are provided in Table 5.

Three types of MPs were identified in the neonatal blood samples, with polyethylene (PE) being the most abundant, followed by polyvinyl stearate (PVS), accounting for 95.65% (88/92) and 3.26% (3/92) of the total detected particles, respectively. MPs spanning 12 distinct size ranges were identified across the samples.

### 3.4 Comparison of microplastic particle abundance in neonatal blood among the three groups

**Figure 2:**
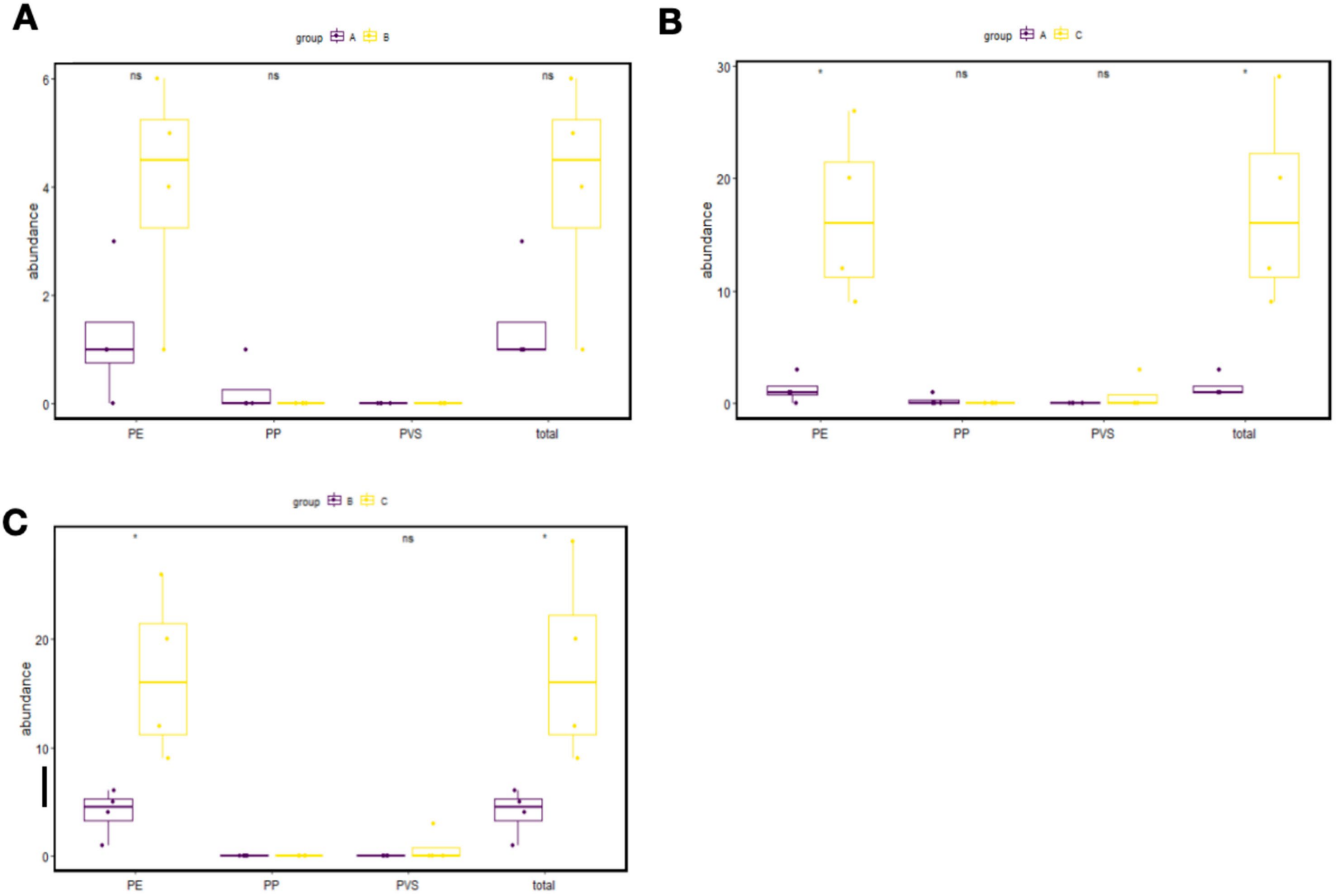
Comparison of MP particle numbers between the short-term exposure group and control group **(A)**, between the long-term exposure group and control group **(B)**, and between the long-term exposure group and short-term exposure group **(C)**. PP, polypropylene; PE, polyethylene; PVS, polyvinyl chloride.

The comparison of particle counts for different MP types among the exposure groups is presented in Figure 2. No statistically significant difference was observed in MP particle counts between the short-term exposure group and the control group (*p* > 0.05).

In contrast, the long-term exposure group exhibited significantly higher total MP particle counts compared to both the control group (*p* < 0.05) and the short-term exposure group (*p* < 0.05). This elevation was predominantly driven by an increase in polyethylene (PE) particles.

### 3.5 Comparison of microplastic particle diameters in neonatal blood among the three groups

**Figure 3:**
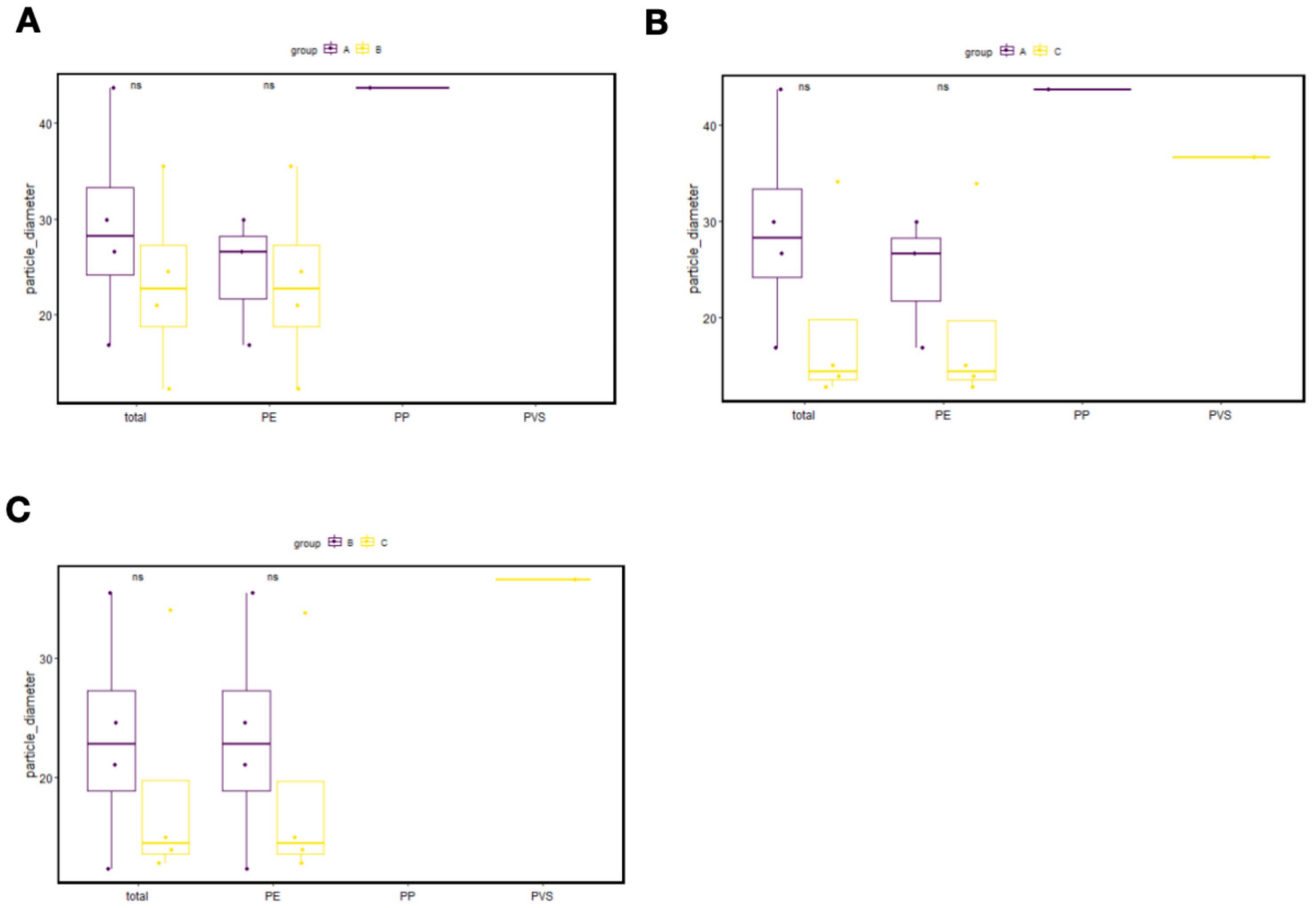
Comparison of MP particle size between the short-term exposure group and control group **(A)**, between the long-term exposure group and control group **(B)**, and between the long-term exposure group and short-term exposure group **(C)**. PP, Polypropylene; PE, Polyethylene; PVS, polyvinyl stearate.

The comparison of particle diameters for different MP types among the exposure groups is presented in Figure 3. No statistically significant difference was observed in the overall MP size distribution among the three groups (*p* > 0.05).

Notably, despite overlapping size ranges, MPs with larger diameters (>60 μm) were exclusively detected in the long-term exposure group. This observation suggests that prolonged PN infusion may facilitate the entry of larger plastic particles into the systemic circulation, lending support to the hypothesis that intravenous administration can bypass physiological barriers, allowing direct translocation of MPs into the bloodstream.

## 4 Discussion

Through a prospective gradient exposure cohort, this study has, for the first time, systematically revealed a unique and serious iatrogenic exposure scenario in the NICU: systemic contamination of neonates with MPs originating from the PN infusion system. Our findings provide compelling evidence that neonates receiving PN support have detectable MPs in their bloodstream, and the body burden shows a significant dose–response relationship with the duration of PN. Neonates in the long-term exposure group (PN > 14 days) exhibited significantly higher MP abundance in their blood compared to both the short-term exposure group and the control group, clearly implicating the PN delivery system as a continuous and potent source of MP exposure. This discovery holds important implications for understanding the additional environmental stressors faced by the most vulnerable infants in the NICU.

The MPs identified in neonatal blood were predominantly polyethylene (PE), accounting for 95.65% of the total. This polymer profile aligns closely with the global plastic production landscape and the common materials used in medical devices (14–17), strongly supporting the hypothesis that the PN infusion system is the primary contributor to MPs in neonatal circulation.

Notably, while bottle-feeding has been considered a potential source of MPs for infants, MPs were detected in some bottle-feeding samples in this study, their abundance was substantially lower than in blood samples. More importantly, no MPs were detected in any breast milk samples. This contrast further strengthens our inference that intravenous infusion constitutes a highly efficient exposure pathway that bypasses all gastrointestinal physiological barriers, leading directly to systemic circulation. The resulting internal exposure level may far exceed that of oral ingestion.

A central finding of this study is the clear dose–response relationship between MP burden and PN duration. The MP particle count in the long-term exposure group was significantly higher not only than that in the unexposed control group but also compared to infants receiving only short-term PN. This indicates that the duration of PN therapy is a critical risk factor in determining the cumulative load of MPs in neonates.

In clinical practice, infants requiring long-term PN are often the most vulnerable, including those who are extremely preterm, have an extremely low birth weight, or suffer from severe intestinal diseases (18, 19). Consequently, they face a compounded risk from MPs on top of their existing clinical complications, raising significant health concerns.

Another noteworthy finding pertains to the size distribution of MPs. Although no statistically significant difference was observed in the overall size distribution among the three groups, individual MPs with relatively larger diameters (>60 μm) were observed in the long-term exposure group. Traditional toxicology posits that MPs capable of crossing biological barriers (e.g., intestinal wall and alveoli) are primarily nano- or sub-micron-sized (20–22). However, our results suggest that under the “direct” exposure mode of intravenous administration, larger MPs may also directly enter the bloodstream.

These findings challenge previous *in vivo* translocation models based on oral or inhalation exposure and imply that iatrogenic exposure may pose unique risks, including physical hazards (e.g., potential microvascular occlusion and direct mechanical damage to blood cells) and chemical hazards (larger particles have a greater surface area for leaching additives).

The unique physiology of neonates may render them more susceptible to MPs. Their immature immune systems, underdeveloped detoxification and metabolic organs, and incomplete protective structures, such as the blood–brain barrier (23, 24), mean that even low-dose MP exposure could potentially interfere with normal growth and development or induce long-term health issues (25, 26). MP exposure has been associated with adverse outcomes such as growth restriction and metabolic disturbances (27–29).

Our study is the first to link this exposure risk to a specific, clinically essential iatrogenic route, highlighting the severity of the issue.

While revealing the risk of PN-associated iatrogenic MP exposure, this study urgently calls for a transition from “risk identification” to “risk prevention and control” in practice. To achieve this, future efforts should promote the establishment of a comprehensive protection framework encompassing clinical management, industry standards, and health education.

At the NICU clinical level, it is recommended to clearly identify neonates on long-term PN as a high-risk group. Management strategies should be refined based on MP release data across different tubing materials, prioritizing low-release materials, standardizing replacement cycles, and exploring medical-grade in-line filters.

At the industry standard level, there is an urgent need for collaboration among materials science, clinical medicine, and regulatory bodies to jointly establish safety limits and evaluation standards for MP release from critical medical consumables such as infusion tubing, thereby controlling contamination at the source.

At the level of public awareness, targeted educational campaigns on iatrogenic MP protection should be conducted for healthcare professionals and parents of newborns to raise risk awareness and guide protective behaviors, translating research findings into tangible clinical and societal actions to protect vulnerable neonates.

Despite these important findings, this study has limitations. First, the sample size was relatively small, which, while common in exploratory studies and sufficient to demonstrate significant inter-group differences in this case, necessitates validation of generalizability in larger, multi-center cohorts.

Second, we were unable to simultaneously collect and analyze the PN solutions being infused and the tubing through which they flowed, thus precluding the establishment of an absolute quantitative link from the contamination source to the internal body burden—a critical aspect for future research to address.

This study focused on exposure assessment; the immediate and long-term health impacts of MPs on neonates (e.g., whether they exacerbate the inherent inflammatory state of preterm infants, affect liver/kidney function, or interfere with growth/neurodevelopment) remain major unanswered questions that urgently require elucidation through long-term follow-up and in-depth mechanistic studies.

## 5 Conclusion

In this exploratory study with a limited sample size, we observed a preliminary association between prolonged exposure to PN and an increased burden of MPs—predominantly polyethylene—in neonatal blood.

The exclusive presence of larger particles (>60 μm) in the long-term PN group further implies that intravenous delivery may facilitate the direct entry of MPs into the systemic circulation, bypassing gastrointestinal barriers.

However, due to the small cohort, these findings should be interpreted with caution and do not conclusively establish the PN system as a direct source of MPs. Rather, they serve as hypothesis-generating evidence that underscores the need for large-scale, multi-center investigations to validate these observations and assess the potential long-term health implications for this vulnerable population.

## Data Availability

The datasets presented in this study can be found in online repositories. The names of the repository/repositories and accession number(s) can be found in the article/supplementary material.
